# The Arabidopsis ATP-BINDING CASSETTE Transporter ABCB21 Regulates Auxin Levels in Cotyledons, the Root Pericycle, and Leaves

**DOI:** 10.3389/fpls.2019.00806

**Published:** 2019-06-19

**Authors:** Mark K. Jenness, Nicola Carraro, Candace A. Pritchard, Angus S. Murphy

**Affiliations:** ^1^Department of Plant Science and Landscape Architecture, University of Maryland, College Park, MD, United States; ^2^Department of Horticulture and Landscape Architecture, Purdue University, West Lafayette, IN, United States

**Keywords:** ABCB transporter, *Arabidopsis thaliana*, auxin, development, seedling

## Abstract

The phytohormone auxin plays significant roles in regulating plant growth and development. In Arabidopsis, a subset of ATP-BINDING CASSETTE subfamily B (ABCB) transporters participate in polar movement of auxin by exclusion from and prevention of reuptake at the plasma membrane. A previous analysis identified ABCB21 as a conditional auxin uptake/efflux transporter that regulates cellular auxin levels, but clear physiological roles for ABCB21 *in planta* remain unknown. Here we show that ABCB21 maintains the acropetal auxin transport stream by regulating auxin levels in the pericycle. Loss of ABCB21 reduces rootward auxin transport and delays lateral root emergence. In seedling shoots, ABCB21 regulates mobilization of auxin from the photosynthetic cotyledons that is important for phototropic bending. In rosette leaves ABCB21 contributes to lateral auxin distribution. These results support a primary role for ABCB21 in regulating auxin distribution supplementary to the primary ABCB auxin transporters ABCB1 and 19.

## Introduction

Optimization of light capture from sunlight and accumulation of water and nutrients from the soil during seedling establishment are major determinants of plant fitness. In Arabidopsis seedlings, light promotes synthesis of the phytohormone auxin (indole-3-acetic acid, IAA) in the cotyledons and young developing leaves (Bhalerao et al., 2002). During early post-photomorphogenic seedling growth auxin regulates expansion of the photosynthetically active cotyledons (Ni et al., 2001; Lewis et al., 2009) and tropic bending toward the light source (Christie et al., 2011). During later stages of seedling growth auxin regulates lateral root development in a sequence of events that can be grouped into two distinct major phases: initiation and elongation/emergence (Péret et al., 2009). Auxin originating from or redirected at the root cap controls transcriptional oscillations that initiate lateral root primordia (Van Norman, 2015; Xuan et al., 2015) within the xylem pole pericycle which surrounds the central vascular cylinder (Dolan et al., 1993; Dubrovsky et al., 2000; Casimiro et al., 2001; Van Norman et al., 2013; Kircher and Schopfer, 2016). Excision and labeling experiments in Arabidopsis demonstrate that auxin involved in both lateral root initiation and emergence is initially shoot derived and transported in a rootward stream between 5 and 7 d after germination (Busse and Evert, 1999; Bhalerao et al., 2002; Lewis et al., 2007; Swarup et al., 2008; Peer et al., 2014). The requirement for auxin synthesis at the root apex as seedlings mature was more definitively demonstrated when root growth of quadruple *yucca* auxin biosynthesis mutants was rescued by exogenous auxin in the media, but not by auxin overproduction in the shoot (Chen et al., 2014). Further, although shoot-derived auxin induces lateral root emergence, local auxin biosynthesis in the root tip is also required for root meristem maintenance (Brumos et al., 2018). At 10 days post-germination the root apex increases competence to synthesize auxin and, subsequently, root-derived auxin maintains primary root growth (Bhalerao et al., 2002; Brumos et al., 2018).

The rootward polar auxin stream in seedlings is primarily attributed to a cellular transport process that involves gradient-driven, directed release to the apoplast of auxin from one cell followed by uptake into an adjoining cell. Bulk auxin movement in phloem transport makes an additional contribution to movement as seedlings mature (Swarup et al., 2001; Marchant et al., 2002). At the cellular level, isotropic auxin (IAA) uptake occurs via lipophilic diffusion of the protonated acid or H^+^ symport of the prevalent anionic form via AUXIN RESISTANT1/LIKE AUX1 (AUX1/LAX) permeases. AUX1/LAX proteins play a primary role in auxin redirection at the root apex and uptake into cortical cells during lateral root emergence (Bennett et al., 1996; Swarup and Péret, 2012). Polarized PIN-FORMED (PIN) proteins facilitate directional cellular efflux vectors to amplify overall polar streams (reviewed in Adamowski and Friml, 2015), while the activity of ATP-BINDING CASSETTE subfamily B (ABCB) efflux transporters limits auxin reuptake at efflux sites (Blakeslee et al., 2007; Aller et al., 2009; Bailly et al., 2011).

Observations of cellularly-polarized PIN proteins that function in organogenic growth by amplifying vectoral auxin streams (Benková et al., 2003; Friml et al., 2003) harmonize well with predictions of early polar auxin transport models (Rubery and Sheldrake, 1974; Raven, 1975; Goldsmith, 1977). Polar transport defects evident in mutants where transport sinks generated by AUX1/LAX uptake are absent (Bennett et al., 1996; Marchant et al., 1999; Swarup et al., 2001; Péret et al., 2012) are consistent with a requirement for uptake sinks included in more robust models (Lomax et al., 1995; Kramer and Bennett, 2006). Finally, alterations in plant stature, changes in leaf morphology, and reductions in long distance polar auxin streams associated with loss of ABCB function (Noh et al., 2001; Multani et al., 2003; Geisler et al., 2005; Santelia et al., 2005; Terasaka et al., 2005; Blakeslee et al., 2007; Knöller et al., 2010) are consistent with cellular efflux models that include cellular exclusion at the PM interface (Bailly et al., 2011; Jenness and Murphy, 2014). These later models factor in membrane partitioning of auxin (Gutknecht and Walter, 1980) and direct binding of ABCB transporters with the auxin efflux inhibitor 1-naphthylphthalamic acid (NPA) (Noh et al., 2001; Murphy et al., 2002; Geisler et al., 2003; Bernasconi et al., 2016), as well as experimentally-determined losses of rootward auxin transport (60–75% in Arabidopsis *abcb1 abcb19* seedlings (Blakeslee et al., 2007).

Except during cell division, ABCB proteins exhibit nonpolar distributions on the plasma membrane (PM) (Geisler et al., 2005; Blakeslee et al., 2007; Wu et al., 2007; Mravec et al., 2008; Kubeš et al., 2012). Accordingly, *ABCB* mutants are competent in embryo- and organogenesis, but exhibit vegetative phenotypes indicative of reduced and irregular cell elongation/expansion (Noh et al., 2001; Wu et al., 2007). In almost all plant species studied, a highly similar pair of ABCB proteins (ABCB1 and 19 in Arabidopsis) are primary contributors to rootward auxin transport (Knöller et al., 2010). In maize and other grasses, ABCB1/Brachytic2/Dwarf3 is a primary regulator of rootward auxin transport (Multani et al., 2003; Cassani et al., 2010; Knöller et al., 2010; McLamore et al., 2010; Balzan et al., 2018; Wei et al., 2018). In Arabidopsis and other dicots, ABCB19 is the more distinguishable isoform, and loss of ABCB19 results in enhanced phototropic bending (Noh et al., 2003; Christie et al., 2011), reduced plant stature (Noh et al., 2001), decreased auxin reporter activity in early stage lateral roots, and reduced lateral root outgrowth (Wu et al., 2007). The additional contribution of ABCB1 to rootward streams is best visualized in *abcb1 abcb19* double mutants (Lin and Wang, 2005; Blakeslee et al., 2007; Wu et al., 2007).

However, the Arabidopsis genome encodes 22 full-length ABCB transporters, including the pseudogene *ABCB8* (Verrier et al., 2008). Some of these isoforms appear to function in localized maintenance of rootward auxin transport streams, as treatment of Arabidopsis seedlings with the ABCB-associated auxin transport inhibitors NPA, Gravacin, and BUM (2-[4-(diethylamino)-2-hydroxybenzoyl benzoic acid) causes delayed lateral root formation and emergence to a greater extent than is observed in *abcb1 abcb19* mutants alone (Casimiro et al., 2001; Rojas-Pierce et al., 2007; Kim et al., 2010). Recently, the ABCB6 and ABCB20 auxin transporters were shown to contribute to rootward auxin streams in inflorescences (Zhang et al., 2018), and the biochemically uncharacterized ABCB11/12 pair, guard cell malate/citrate transporter ABCB14 (Lee et al., 2008), and ABCB15 in the Arabidopsis inflorescence have also been implicated in maintenance of rootward auxin streams (Kaneda et al., 2011).

A contribution of ABCB21 to acropetal auxin streams in the root has also been inferred by localization of *proABCB21:GUS* signals to the root vasculature and biochemical characterizations of conditional auxin transport in protoplasts and yeast exhibiting attributes that are highly similar to the root epidermal/cortical ABCB4 transporter (Kamimoto et al., 2012). This suggests that ABCB21 functions in the vascular cylinder of the root maturation zone and above to provide a regulated lateral boundary for the rootward auxin transport stream. Such function would require a conditional auxin uptake/efflux transport activity in the pericycle of the maturation zone. This function is hypothesized to be similar to ABCB4 modulation of constitutive shootward auxin transport from the root apex mediated by AUX1 and PIN2 in epidermal cells near the root elongation zone (Santelia et al., 2005; Terasaka et al., 2005; Cho et al., 2007; Yang and Murphy, 2009; Kubeš et al., 2012). Additionally, ABCB21 expression in young leaves suggests an analogous function in those organs (Kamimoto et al., 2012).

Here we show that ABCB21 maintains the acropetal auxin transport stream by regulating auxin levels in the pericycle and functions in the distribution of auxin in cotyledons and young leaves. Loss of ABCB21 results in reduced rootward auxin transport and defects in lateral root outgrowth. In aerial tissues, *abcb21* exhibits reduced cotyledon-hypocotyl auxin transport, defects in phototropic bending, and alterations in lateral auxin movement in leaves. While the exclusionary role of ABCB21 supplements the activity of ABCB19, the conditional uptake/efflux activity provides an additional and unique level of auxin transport regulation. Sequence similarity implies functional redundancy between the ABCB4/21 pair, as is observed with ABCB1/19 and ABCB6/20 (Noh et al., 2001; Zhang et al., 2018). Unlike these pairs, ABCB4 and ABCB21 function in discrete domains, indicating distinct spatio-temporal roles during growth and development.

## Materials and Methods

### Plant Material and Growth Conditions

*Arabidopsis thaliana* ecotype Columbia (Col-0) was used for all experiments. Lines used are listed in Supplementary Table 3. Seeds were surface sterilized and sown on 1/4 MS medium (pH 5.6; Caisson Labs, Smithfield, UT, USA) containing 1 g L^−1^ MES, 0.5% sucrose, and 0.8% agar, pH 5.5. For seedling assays, seeds were stratified 4°C for 2 d, then grown vertically under continuous 100 μmol m^−2^ s^−1^ light at 22°C for the times indicated. For mature plants, seeds were sown on soil, stratified 4°C for 2 d, then grown in growth chambers under 100 μmol m^−2^ s^−1^ light (16 h photoperiod) at 22°C for the times indicated.

### Yeast Transport Assays

Yeast assays were conducted as described (Yang and Murphy, 2009). The ABCB21 expression construct was created by amplifying the *ABCB21* coding sequence with Gateway BP primers (Supplementary Table 4) and recombining the product into pDONR/Zeo by BP reaction (Thermo Fisher Scientific). *ABCB21* was then transferred into pREP41GW by LR reaction (Thermo Fisher Scientific). Expression vectors were transformed into *S. pombe* by electroporation. Assays were performed using 40 nM [^3^H]IAA, which is within the physiological range for Arabidopsis (Novák et al., 2012).

### Histochemical Staining

The 0.625 kb promoter fragment of *ABCB21* upstream of the start codon was cloned into pENTR/D-TOPO (Thermo Fisher Scientific) then transferred into the Gateway compatible vector pGWB3 (Nakagawa et al., 2007) by LR reaction (Thermo Fisher Scientific). Constructs were transformed into Col-0 via floral dip (Clough and Bent, 1998). For GUS staining, tissues were incubated in 90% acetone for 20 min on ice, then immersed in staining solution (50 mM sodium phosphate buffer (pH 7.0), 0.1% Triton X-100, 0.5 mM potassium ferrocyanide, 0.5 mM potassium ferricyanide, and 1 mM X-gluc) and incubated in the dark at 37°C for 5 h, unless otherwise noted. Stained samples were cleared with 70% ethanol before imaging. For sectioning tissue was dehydrated in a series of tert-butanol (TBA) and embedded in Paraplast Plus. Twenty micrometer sections were prepared using a Leica Reichert-Jung 2030 rotary microtome.

### Seedling Transport Assays

Rootward seedling transport assays were conducted as described (Christie et al., 2011), except that 6% agarose beads (Colloidal Science Solutions; AMB-0601-0010) were substituted for polystyrene beads. For cotyledon-hypocotyl transport assays 5 d seedlings were placed on filter paper (Whatman 3MM) saturated with 1/4 MS with the hypocotyl and cotyledons not touching any surface. Seedlings were allowed to equilibrate vertically in light for 1 h. A 6% agarose bead incubated in solution containing 2 μM IAA (1:1 cold IAA:[^3^H]IAA; 25 Ci mmol^−1^, American Radiolabeled Chemicals) was placed in the middle of one cotyledon per seedling. After 2 h, both cotyledons were removed by cutting just below the cotyledonary node using a surgical blade. [^3^H]IAA transported from the cotyledons to the hypocotyl and root was measured by liquid scintillation counting.

### Leaf Transport Assays

Agarose beads coated in [^3^H]IAA were placed on equal size rosette leaves of 35 d plants at the positions indicated. After bead placement plants were incubated under 20 μmol m^−2^ s^−1^ yellow light and 55% relative humidity for 3 h. 0.5 mm punches were collected at positions indicated and measured for radioactivity.

### IAA Quantifications

Free IAA quantifications were conducted as described (Novák et al., 2012) with minor modifications. Briefly, 10–16 mg Arabidopsis tissue was collected and frozen in liquid nitrogen before storing in −80°C until use. Samples were ground in liquid nitrogen, and 1 mL cold 50 mM sodium phosphate buffer (pH 7.0) containing 1% diethyldithiocarbamic acid. Indole proprionic acid was added as an internal standard. Samples were vortexed and extracted for 20 min at 4°C, then centrifuged at 12,000 × g for 15 min at 4°C. The pH value of supernatant was adjusted to 3 using 1 N HCl, and the supernatants were purified using an HLB Column. The column was conditioned with 1 mL methanol (Fisher Scientific, LC-MS/MS grade, A456-1) followed with 1 mL water and 0.5 mL 50 mM sodium phosphate buffer, pH 2.7. After loading the sample, the column was washed with 2 mL 5% (vol/vol) methanol. Finally, analytes were eluted with 2 mL 80% (vol/vol) methanol. The eluted samples were dried under nitrogen gas, dissolved with 500 μL methanol, and filtered through 0.2-μm PTFE Filters (Fisher Scientific, 03–391-4E); 1 μL of each sample was injected for LC-MS/MS analyses. Compounds were quantified in positive ion mode, and MS/MS settings were as described (Novák et al., 2012) and conducted by Agilent 6460 triple quadrupole LC-MS/MS.

### RNA Isolation and Quantitative Real-Time PCR (qRT-PCR)

For qRT-PCR total RNA was extracted using ZR Plant RNA Mini Prep kit (Zymo Research) followed by treatment with DNasel (New England Biolabs). Total RNA (1.5 μg) was used for first-strand synthesis using SuperScript III reverse transcriptase (Thermo Fisher Scientific). qRT-PCR was performed on a CFX Connect (Bio-Rad Laboratories) using EvaGreen qPCR master mix (Biotium) according to manufacturer's instructions. Primers used are listed in Supplementary Table 4. Transcript levels normalized against *PP2A* (AT1G69960) or *ACT2* (AT3G18780) produced similar results.

### Phototropism Assays

Surface sterilized seeds were sown on MS, 0.5% sucrose, and 0.8% agar plates then stratified at 4°C for 2–4 d in the dark. To induce germination seeds were placed in light for 12 h. For etiolated bending assays seeds were removed from light and placed in dark for 3 d at 22ÂC until hypocotyls reached 7–8 mm in height. For de-etiolated bending assays seeds were placed in light ~36 h to undergo photomorphogenesis, then place in dark to induce hypocotyl elongation to 7–8 mm in height. 3 d seedlings were transferred from vertical plates to 60 mm petri dishes filled with silicon dioxide (Sigma, St. Louis, MO; Cat #274739) and water. After a 30 min acclimation, seedlings were exposed to 0.4–0.8 μmol m^−2^ s^−1^ unilateral LED blue light illumination. Images were captured every 10 min with a USB3 uEYE CP camera (IDS Imaging Development Systems, Woburn, MA, USA) and processed for clarity with Photoshop (Adobe Systems Inc., Cupertino CA). All phototropic bending and hypocotyl elongation measurements were made using FIJI software (Schindelin et al., 2012; Schneider et al., 2012). Bending angle measurements are described in Supplementary Figure 6.

### Leaf Petiole Angle Assays

Assays were conducted as described (de Carbonnel et al., 2010). Briefly, soil was placed in 90 ×15 mm petri dishes with holes punched in the bottom. Dishes were then placed in trays and watered by bottom infiltration. Seeds were sown onto the soil and stratified for 48 h. Seedlings were grown in a growth chamber under 80 μmol m^−2^ s^−1^ white light, 16 h photoperiod. Upon reaching stage 1.01, seedlings were transferred to continuous 50 μmol m^−2^ s^−1^ white light or 50 μmol m^−2^ s^−1^ red light for an additional 72 h. For true leaf petiole angle measurements plants were photographed from the side. Angles were determined by measuring the angle formed between the hypocotyl and the petiole minus 90°.

### Petal Break-Strength Assays

Plants that were grown 35 d at 21°C temp with 16 h 100 μmol m^−2^ s^−1^ light/8 h dark. Detached flowers were suspended on a force transducer (Aurora Scientific, Inc.: Model 404A: range, 0–100 mN; sensitivity; 10.0 mN; resolution, 2000 nN) with an alligator clip lined with soft rubber (Supplementary Figure 7A). Single flower petals were then attached to flat end forceps mounted on a computer controlled translation stage (Thor Labs OptoDC Servo Motor) programmed to move 0.05 mm s^−1^. The vertical displacement of the stage resulted in reproducible detachment of the petal at the receptacle (Supplementary Figure 7B). The maximum voltage value from the acquisition during petal pulling was used to calculate the petal break-strength. The measurements were converted to gram equivalents according to a linear standard curve (voltage as a function of weight) corrected for the weight of the clip (Supplementary Figure 7C). Flowers 2, 4, and 6 were measured with position 1 considered to be the first flower with visible petals.

### Lignin Content and *p*-coumaryl Alcohol Growth Assays

Analysis of lignin thioacidolysis products and *p*-coumaryl alcohol growth assays were conducted as described (Alejandro et al., 2012).

### Statistical Analysis

All statistical analyses were performed using JMP PRO 13.

## Results

### ABCB21 Exhibits Conditional Auxin Uptake/Efflux Activity

**Figure 1:**
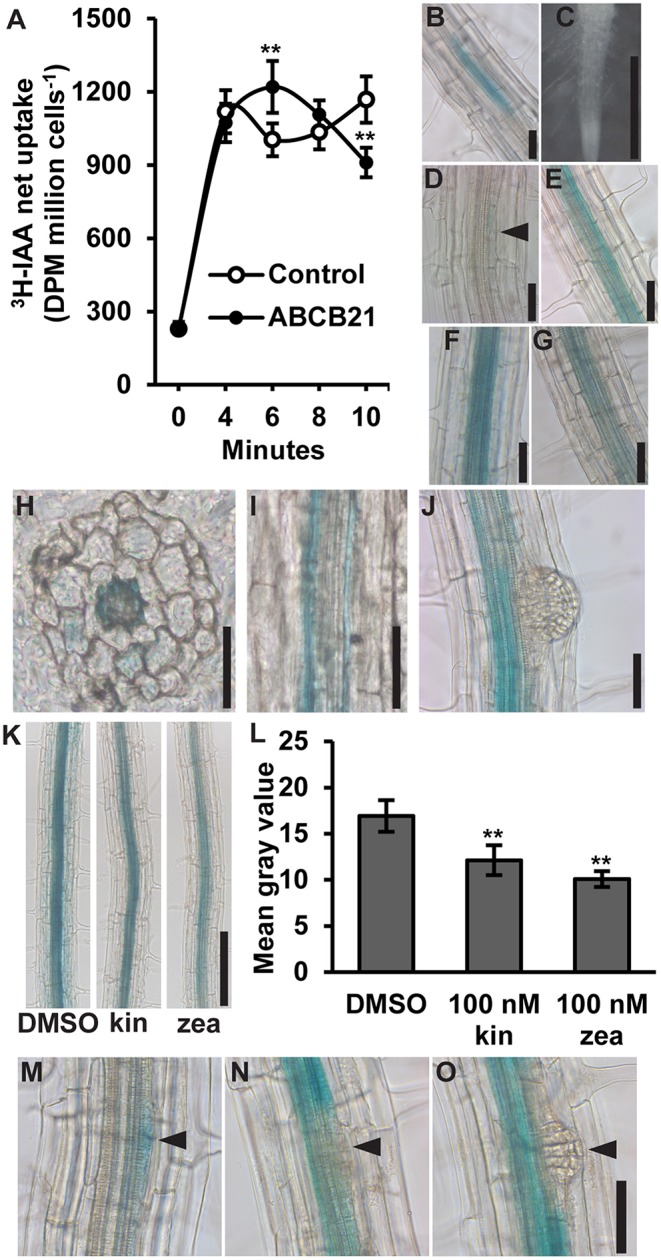
Auxin transport activity and expression of *ABCB21*. **(A)** ABCB21 exhibits conditional auxin uptake/efflux in *S. pombe*. Data shown are means ± SD (*n* = 6–8 from two experiments with 3- independent transformants). **(B)** *proABCB21:GUS* expression in 4 d etiolated seedling roots. **(C–G)** *proABCB21:GUS* expression in **(C)** 1 d **(D)** 3 d, **(E)** 5 d, **(F)** 7 d, and **(G)** 10 d light-grown seedling roots. No GUS signal is observed in 1 d roots. Arrow indicates area of low level expression in 3 d roots. **(H)** Cross and **(I)** longitudinal sectioning confirms expression is primarily associated with the root pericycle. **(J)** *proABCB21:GUS* is absent in emerging lateral roots. **(K)** Treatment with cytokinins reduces *proABCB21:GUS* expression in the pericycle of 7 d roots. Representative images of *proABCB21:GUS* expression after 3 h treatment with 100 nM kinetin (kin) or *trans*-zeatin (zea) prior to GUS staining. **(L)** Quantification of *proABCB21:GUS* signal in **(K)**. Images were taken between the lowest two emerged lateral roots. Data shown are means ± SD for the five most heavily stained roots. **(M–O)** *proABCB21:GUS* expression during early lateral root development. Arrows indicate lateral root primordia. Asterisks indicate statistical difference from Col-0 by Student's *t*-test for ** *P* < 0.01. Scale bars: **(B–J,M–O)** 50 μm; **(K)** 200 μm.

ABCB21 was previously shown to exhibit conditional uptake/efflux activity using *RNAi* knockdown in mesophyll protoplasts and expression in *Saccharomyces cerevisiae* (Kamimoto et al., 2012). This activity was validated by expressing ABCB21 in *Schizosaccharomyces pombe* (Figure 1A). Cells expressing ABCB21 accumulated ~25% more [^3^H]IAA after 6 min than control lines. However, after 10 min cells expressing ABCB21 accumulated ~23% less [^3^H]IAA than the controls. The decrease in IAA accumulation in control lines between 4 and 6 min is indicative of efflux by endogenous low affinity IAA transporters which is not observed until higher cellular IAA levels are reached (Yang and Murphy, 2009). Short timeframes is one of the limitations with using this assay system. After ~10 min the results become more variable and difficult to interpret due to endogenous transport activities and reduced integrity of the yeast cells. These results suggest that, like ABCB4, ABCB21 exhibits initial IAA uptake activity and efflux is activated by reaching a threshold intracellular IAA concentration. The timing of the switch between uptake and efflux is very similar between ABCB21 and those observed for ABCB4 (Yang and Murphy, 2009), suggesting they may share similar transport and/or regulatory mechanisms.

### *ABCB21* Expression in Seedlings Is Associated With Auxin Conducting Tissues

*ABCB21* expression was previously analyzed using a promoter sequence 0.75 kb upstream of the *ABCB21* start codon fused to the β*-glucuronidase* (*GUS*) reporter (Kamimoto et al., 2012). This promoter, however, included 122 nucleotides of the 3′ UTR of the upstream gene (At3g62160). To see if this fragment had any effect on expression, a shorter 0.625 kb promoter was fused to *GUS* and transformed into Col-0. Overall, there were no observable expression differences between the two promoters. In the root, *proABCB21:GUS* is primarily expressed in the root vasculature of 5–10 d seedlings (Figures 1B–G). Before 5 d and after 10 d, expression in the pericycle is mostly absent and the signal that is present is highly variable and discontinuous (Figures 1B–D,G). Cross and longitudinal sections of roots show that expression is primarily associated with the pericycle (Figures 1H,I). Expression in the pericycle is continuous throughout the top two-thirds of the root, but absent in lateral root primordia and emerging lateral roots (Figure 1J). Negative regulation of *ABCB21* expression by cytokinin has been reported in transcriptomic studies (Winter et al., 2007) and is consistent with the observed lack of expression in the root tip where cellular cytokinin levels are high (Antoniadi et al., 2015). Treatment with 100 nM kinetin or *trans*-zeatin (similar to levels reported in the root tip) reduced *proABCB21:GUS* expression in root pericycle cells situated between emerged lateral roots (Figures 1K,L). Negative regulation of *proABCB21:GUS* expression in these tissues is consistent with previously reported regions of increased cytokinin signaling visualized with the *TCS:GFP* cytokinin reporter, particularly with exogenous cytokinin treatment (Bielach et al., 2012). This suggests that cytokinin negatively regulates *ABCB21* in immature root tissues, and may also contribute to negative regulation of *ABCB21* during lateral root formation and emergence (Laplaze et al., 2007; Bielach et al., 2012). Since *proABCB21:GUS* expression levels are low in regions of lateral root primordia initiation, visualization of changes following cytokinin treatment were difficult to interpret. However, loss of *proABCB21:GUS* signal is observed following initial cell divisions during primordia initiation (Figures 1M–O), which correlates with the timing of published cytokinin signaling increases during lateral root development (Bielach et al., 2012).

**Figure 2:**
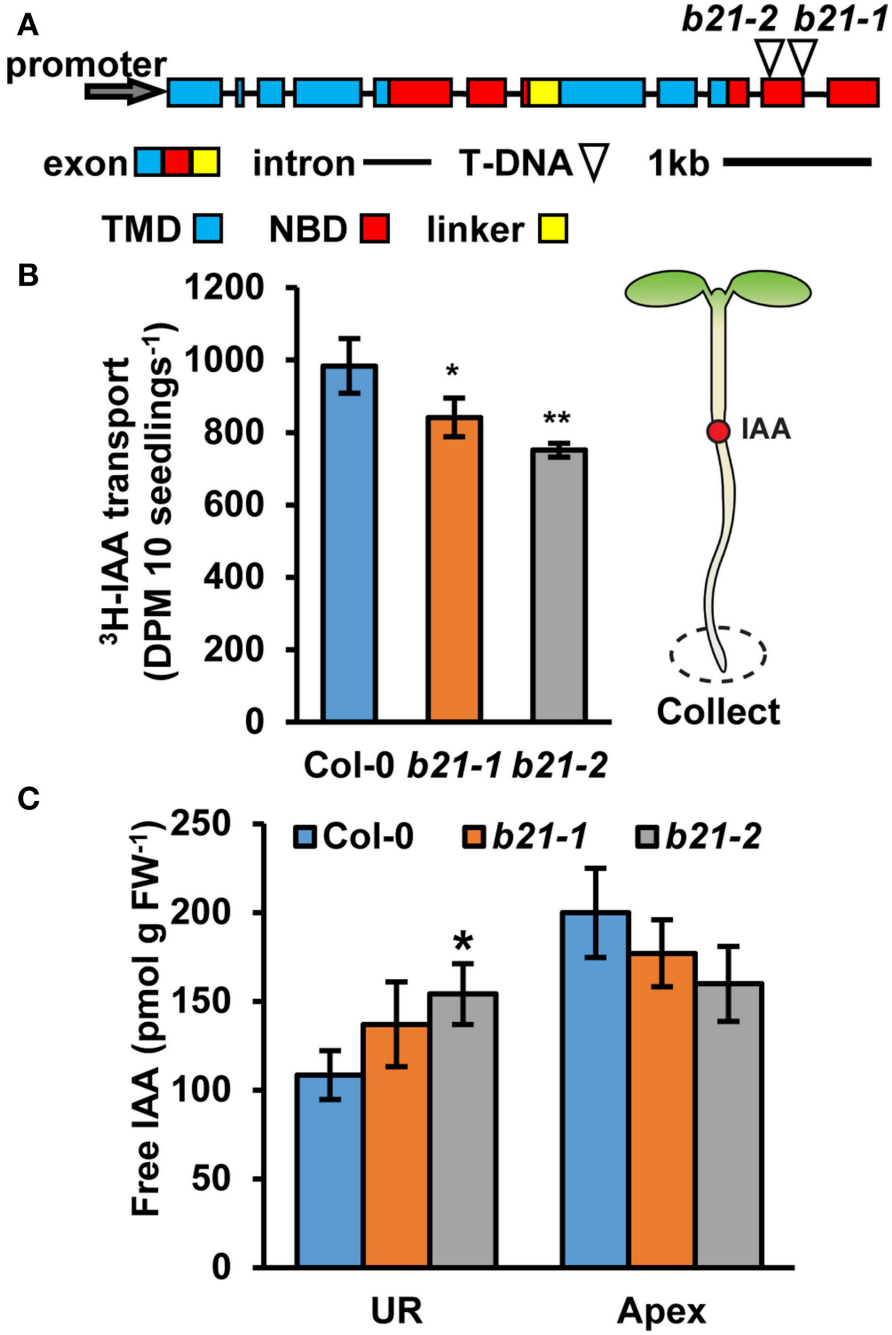
Auxin transport and levels in *abcb21* roots. *abcb21* mutants exhibit reduced acropetal auxin transport in roots. **(A)** *ABCB21* gene model indicating T-DNA insertion positions for *abcb21-1* (WiscDsLox1C2) and *abcb21-2* (Gabi_954H06). Boxes correspond to exons with approximate positions of transmembrane domains (TMD; blue), nucleotide-binding domains (NBD; red), and linker region (yellow). **(B)** [^3^H]IAA transport from the root-shoot transition zone to the root tip in 5.5 d seedlings. Data shown are means ± SD (*n* = 3 pools of 10). **(C)** Quantification of free IAA levels in 5.5 d light grown seedlings. UR, upper root; Apex, apical 2 mm root section. Data shown are means ± SD (*n* = 3 pools of 10). Asterisks indicate statistical difference from Col-0 by Student's *t*-test for * *P* < 0.05 and ** *P* < 0.01.

The previously described *abcb21-1* (WiscDsLox1C2) allele forms a partial transcript (Kamimoto et al., 2012). Therefore, a new allele, *abcb21-2* (Gabi_954H06) was obtained. Mutants were backcrossed to Col-0 three times and genotyped before subsequent analysis (Supplementary Figure 1A). Reverse transcription PCR (RT-PCR) indicates *abcb21-2* also forms a transcript that corresponds to the coding region upstream of the T-DNA insertion (Supplementary Figure 1B). Additionally, expression levels determined by quantitative real-time PCR (qRT-PCR) are not different from Col-0 (Supplementary Figure 1C). However, since the T-DNA insertion in *abcb21-2* is farther upstream compared to *abcb21-1* it was hypothesized that it would represent a stronger allele (Figure 2A). Due to the overlap in expression of *ABCB21* with *ABCB19* surrounding the vasculature, *abcb21-2* was tested for compensation by *ABCB19*. While loss of *abcb1* results in ~5X increase in *ABCB19* transcript, no change is observed in the *abcb21-2* mutant (Supplementary Figure 1D).

**Figure 3:**
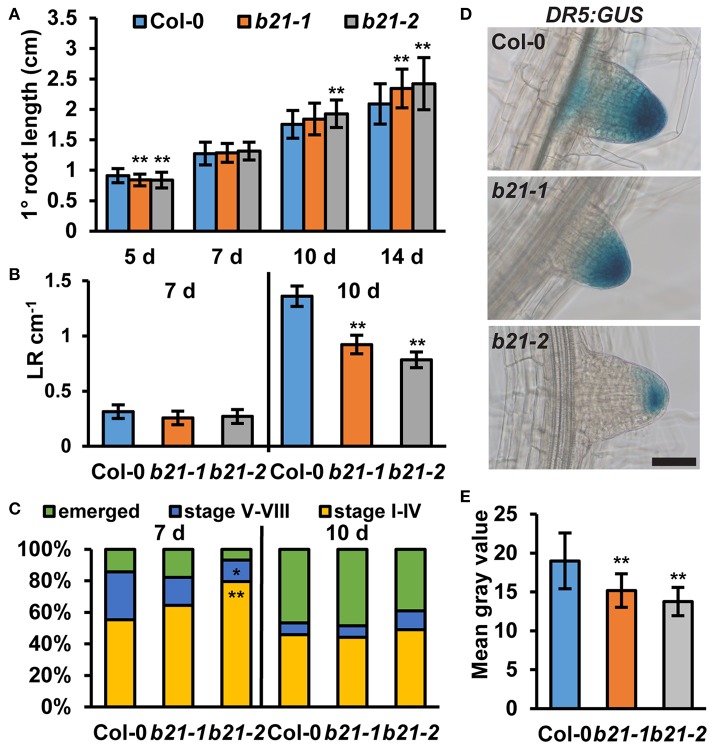
Root phenotypes in *abcb21*. *abcb21* mutants exhibit altered primary root elongation and lateral root development. **(A)** Primary root length at 5, 7, 10, and 14 d. Data shown are means ± SD (*n* = 40–50). **(B)** Emerged lateral root density in 7 and 10 d seedlings. Data shown are means ± SE (*n* = 40–50). **(C)** Distribution of stage I–IV, stage V–VI, and emerged lateral roots in 7 and 10 d seedlings. **(D)** *DR5:GUS* expression is reduced in emerging and newly emerged *abcb21* lateral root tips. **(E)** Quantification of GUS signal in **(D)**. Data shown are means ± SD (*n* ≥ 24 from two independent experiments). Asterisks indicate statistical difference from Col-0 by Student's *t*-test for * *P* < 0.05 and ** *P* < 0.01. Scale bar: 50 μm.

As *ABCB19* and *ABCB21* expression domains overlap in the root, it was hypothesized ABCB21 may function in restriction of auxin to the root vasculature. When [^3^H]IAA was placed at the root-shoot transition zone (RSTZ) transport to the root tip was reduced by ~14% and ~24% in *abcb21-1* and *abcb21-2*, respectively (Figure 2B). Quantification of free IAA levels in *abcb21* indicate auxin levels are increased in the upper root and reduced in the root apex (Figure 2C). This suggests the reduction in rootward auxin transport in *abcb21* causes auxin to back up and pool in the upper root. This pooling, however, was not enough to activate the *DR5:GUS* auxin reporter in either *abcb21* background. Phenotypic analysis revealed *abcb21-2* 5 d primary roots are shorter than Col-0, but slightly longer in 10 and 14 d seedlings (Figure 3A). 10 d *abcb21-2* mutants exhibit reduced lateral root density (Figure 3B), but no difference in the distribution among developmental stages was observed (Figure 3C). Seven days lateral root density is not different between Col-0 and *abcb21* mutants (Figure 3B). However, the proportion of stage I-IV in *abcb21-2* mutants is increased and the proportion of stage V-VIII and emerged lateral roots is reduced in *abcb21-2* (Figure 3C). *abcb21-1* exhibits intermediate primary root and lateral root phenotypes which are consistent with it representing a weak allele compared to *abcb21-2*. *DR5:GUS* signal is reduced in emerging and newly emerged lateral roots indicating the defect in lateral root outgrowth is due to reduced auxin levels (Figures 3D,E).

### ABCB21 Mobilizes Phototropic Auxin Supply From the Cotyledons

**Figure 4:**
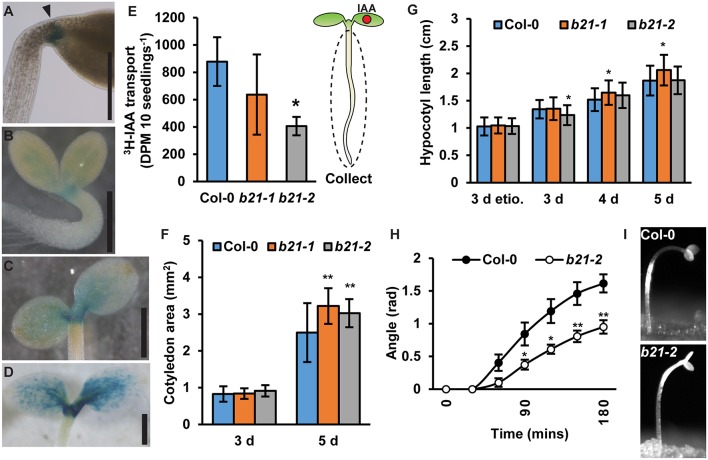
ABCB21 mediates cotyledon-hypocotyl auxin transport. **(A)** *proABCB21:GUS* expression at the base of petioles in 4 d etiolated seedlings. **(B–D)** *proABCB21:GUS* expression in the petioles and cotyledonary node of **(B)** 1 d, **(C)** 3 d, and **(D)** 5 d light-grown seedlings. **(E)** Cotyledon-hypocotyl [^3^H]IAA transport in 5.5 d seedlings. Data shown are means ± SD (*n* = 3 pools of 12). **(F)** Cotyledon areas of 3 and 5 d light-grown seedlings. Data shown are means ± SD (25 ≤ *n* ≤ 32). **(G)** Hypocotyl length in 3 d etiolated (etio.), and 3–5 d light-grown *abcb21* seedlings. Data shown are means ± SD (*n* > 45 from 3 replicates). **(H)** Phototropic curvature in light-treated Col-0 and *abcb21-2* seedlings. Data shown are means ± SE (*n* = 8 from 2 replicates). **(I)** Representative images of Col-0 and *abcb21-2* after phototropic bending for 3 h. Asterisks indicate statistical difference from Col-0 by Student's *t*-test for * *P* < 0.05 and ** *P* < 0.01. Scale bars: **(A–D)** 500 μm.

In seedlings, *proABCB21:GUS* expression is high at the base of the cotyledons, the petioles, and the cotyledonary node (Figures 4A–D) suggesting ABCB21 may function in mobilizing auxin from these tissues. To compare auxin transport in *abcb21* to wild type, [^3^H]IAA was placed at the center of one cotyledon, then the hypocotyl and roots were collected after 2 h (Figure 4E). [^3^H]IAA transport in *abcb21-2* is reduced by >50%. Transport in *abcb21-1* was also reduced, but was highly variable. Auxin transport from the shoot apex to the RSTZ was not different in either *abcb21* mutant (data not shown), which is consistent with the lack of *proABCB21:GUS* expression in the hypocotyl. The defects in mobilization of auxin from the cotyledons leads to a significant increase in cotyledon expansion in 5 d seedlings (Figure 4F), but only small differences in hypocotyl elongation (Figure 4G). Removal of the cotyledons in post-photomorphogenic seedlings reduces phototropic bending, suggesting cotyledon-derived auxin contributes to phototropic bending (Preuten et al., 2013). Similarly, phototropic bending was severely reduced in post-photomorphogenic *abcb21-2* seedlings (Figures 4H,I; Supplementary Movie 1). No difference was observed in etiolated seedlings.

### ABCB21 Contributes to Lateral Auxin Distribution in Rosette Leaves

**Figure 5:**
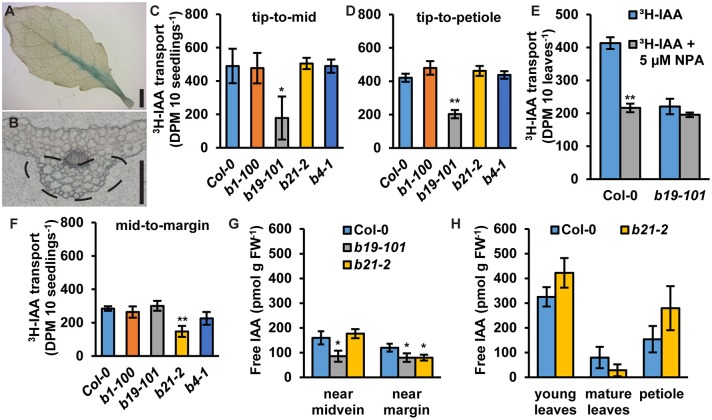
Expression of *ABCB21* and auxin transport activity in leaves. **(A)** *proABCB21:GUS* is expressed in rosette leaves associated with the midvein. **(B)** Cross section showing *proABCB21:GUS* expression is primarily in the bundle sheath and collenchyma on the abaxial side of the leaf. Dotted line indicates area of GUS staining. **(C,D)** Transport of [^3^H]IAA from the leaf tip to the **(C)** leaf midpoint or **(D)** petiole. **(C,D)** share the same scale/units. Data shown are means ± SD (*n* = 3 pools of 10). **(E)** Transport of ^3^H-IAA from the leaf tip to the petiole with NPA treatment. Data shown are means ± SD (*n* = 3 pools of 10). **(F)** Transport of [^3^H]IAA from the leaf midvein to the margin. Data shown are means ± SD (*n* = 3 pools of 10). **(G)** Free IAA levels in rosette leaves near the midvein and near the margin. **(H)** Free IAA levels in young leaves, mature leaves, and petioles. Data shown are means ± SD (*n* = 3 pools of 10). Asterisks indicate statistical difference from Col-0 by Student's *t*-test for * *P* < 0.05 and ** *P* < 0.01. Scale bars: **(A)** 2 mm; **(B)** 200 μm.

In rosette leaves, *proABCB21:GUS* expression is observed near the leaf midvein (Figure 5A). Cross sections revealed that expression is primarily associated with the collenchyma and bundle sheath cells on the abaxial side of the leaf and not within the midvein (Figure 5B). To see if ABCB21 function in the leaf might resemble its function in the root *abcb21-2* mutants were analyzed for defects in auxin transport using intact rosette leaves. *abcb1, abcb19*, and *abcb4* were also included. For transport along the tip-petiole axis, [^3^H]IAA-soaked agarose beads were placed on leaf tips. After 3 h, petioles or 0.5 mm mid-leaf punches were collected and measured for radioactivity. For centro-lateral transport, [^3^H]IAA-soaked agarose beads were placed on the leaf midvein. After 3 h, 0.5 mm punches were collected from the leaf margin and measured for radioactivity. Transport of [^3^H]IAA from the tip to the mid-leaf and petiole was significantly reduced in *abcb19* (Figures 5C,D). In Col-0, treatment with 5 μM NPA reduced transport from the leaf tip to the petiole ~50%, which is equal to the transport in *abcb19* (Figure 5E). Treatment of *abcb19* with 5 μM NPA did not cause any further reduction, suggesting ABCB19 is a primary target for NPA inhibition at this concentration (Figure 5E). No additional reduction in auxin transport from the leaf tip to the midpoint or petiole was observed in Col-0 using 10 μM NPA (Supplementary Figures 2A,B). However, treatment with 20 μM NPA resulted in additional inhibition of transport which is likely due to blocking of other ABCBs and/or PINs. In contrast to *abcb19, abcb21* showed a significant decrease in transport of [^3^H]IAA from the midvein to the margin (Figure 5F). For lateral auxin transport 10 μM NPA inhibited auxin transport in Col-0 to *abcb21* levels (Supplementary Figure 2C). No additional effect was observed using 20 μM NPA (Supplementary Figure 2C) suggesting ABCB21 is the primary target for NPA inhibition of the measured lateral auxin movement. Consistent with these results, endogenous IAA levels are significantly reduced near the midvein of *abcb19* (Figure 5G). IAA levels along the margin are reduced by ~30% in *abcb19* and *abcb21* (Figure 5G). Overall auxin levels in young leaves, mature leaves, and petioles were not statistically different from Col-0 (Figure 5H). Despite the auxin transport defects in leaves, *abcb21* mutants do not exhibit any observable differences from Col-0 in rosette leaf morphology or phyllotaxis under our standard growth conditions (Supplementary Figures 3A,B). It was noted, however, that *abcb21* occasionally exhibited larger variation in rosette leaf size in the greenhouse when light and temperature were more inconsistent.

**Figure 6:**
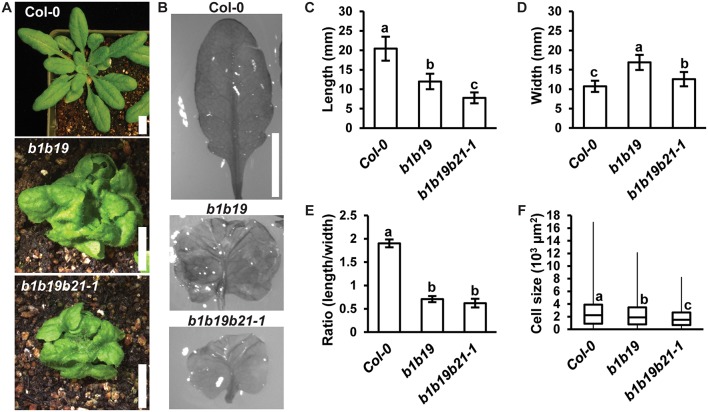
*abcb21* triple mutants exhibit enhanced morphological defects in leaves. **(A)** Representative images of 4 weeks rosettes grown under 100 μmol m^−2^ s^−1^ white light. **(B)** 5th rosette leaf removed from A. Leaves were soaked in ethanol to allow curled leaves to lay flat. **(C–E)** Measurement of **(C)** length, **(D)** width, and **(E)** length/width ratio in leaves from **(B)**. Data shown are means ± SD (*n* ≥ 10). Letters indicate statistical difference by ANOVA *p* < 0.001, Tukey's *post-hoc p* < 0.05. **(F)** Boxplot of showing 5th leaf cell size (*n* ≥ 219 from at least 3 leaves per line). Abaxial epidermal cells were measured at a midpoint from the leaf tip to the petiole and half way from the leaf margin. Letters indicate statistical difference by ANOVA *P* < 0.001, Tukey's *post-hoc P* < 0.05. Scale bars: 1 cm.

Loss of *abcb1* and *abcb19* results in compact and severely curled rosette leaves (Noh et al., 2001; Geisler et al., 2003; Blakeslee et al., 2007). It was hypothesized that addition of *abcb21* would result in enhancement of these leaf morphology defects. Careful examination of leaf development revealed no defects in basic abaxial/adaxial definition, veination, or leaf margin development in *abcb1 abcb19* or *abcb1 abcb19 abcb21* (Figures 6A,B). Although the weak *abcb21-1* allele was used, enhancement of defects in leaf morphology were observed. Measurement of leaf length and width revealed *abcb1 abcb19 abcb21* leaves were significantly shorter than Col-0 and *abcb1 abcb19* (Figure 6C) and wider than Col-0, but not *abcb1 abcb19* (Figure 6D). While triple mutants developed smaller leaves compared to the double mutant, their overall shapes were not notably different (Figures 6B,E). These alterations in morphology appear to be due to reduced abaxial pavement cell expansion, as decreasing cell size compared to Col-0 is observed in *abcb1 abcb19* and *abcb1 abcb19 abcb21*, respectively (Figure 6F).

At flowering stage single *abcb21* mutants are slightly taller than Col-0 plants and have increased secondary inflorescence number (Supplementary Figures 4A–C). No difference in primary branch number or internode length were observed (Supplementary Figures 4D,E). Compensation and functional redundancy lead to enhanced phenotypes in double *abcb1 abcb19* and *abcb6 abcb20* mutants (Noh et al., 2001; Geisler et al., 2003; Blakeslee et al., 2007; Zhang et al., 2018). Although no compensation in *ABCB4* expression was detected in *abcb21* knockdown lines (Kamimoto et al., 2012) and their expression domains do not overlap, *abcb4 abcb21* double mutants were examined for morphological defects not observed in the single mutants. This, however, did not result in any synergistic phenotypes (Supplementary Figures 3A,B, 4A–E).

### *ABCB21* Expression Is Rapidly Induced During Wounding

**Figure 7:**
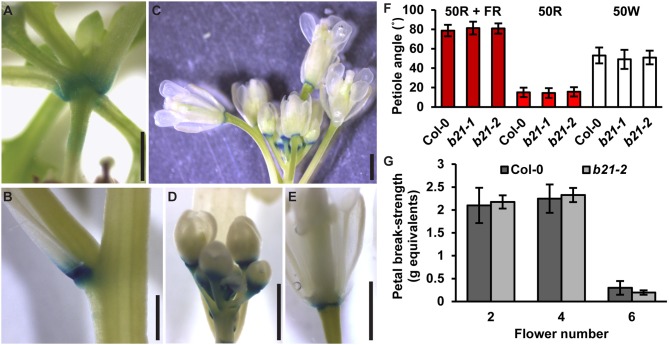
Expression of *ABCB21* in abscission zones. *proABCB21:GUS* is expressed in the abscission zones of **(A)** rosette leaves, **(B)** cauline leaves, and **(C)** floral organs. **(D,E)** *proABCB21:GUS* expression domain is expanded in **(D)** young flowers and **(E)** restricted to the abscission zone in mature flowers. Plants were GUS stained for 16 h. **(F)** True leaf petiole angles in *abcb21*. Plants were grown on soil under 80 μmol m^−2^ s^−1^ white light, 16 h photoperiod. When plants reached stage 1.01 they were transferred to continuous 50 μmol m^−2^ s^−1^ red plus far-red light (50R + FR; burgundy bars), 50 μmol m^−2^ s^−1^ red light (50R; red bars), or 50 m^−2^ s^−1^ white light (50W; white bars) and allowed to grow an additional 3 d. Angle was determined by measuring the angle formed between the hypocotyl and the two first true leaf petioles minus 90°. Data shown are means ± SD (*n* = 60). **(G)** Flower petal break-strength in *abcb21*. Flower 1 was designated as the first flower with visible flower petals. Methods are detailed the methods section and Supplementary Figure 7. Data shown are means ± SD (*n* = 15). Scale bars: 1 mm.

**Figure 8:**
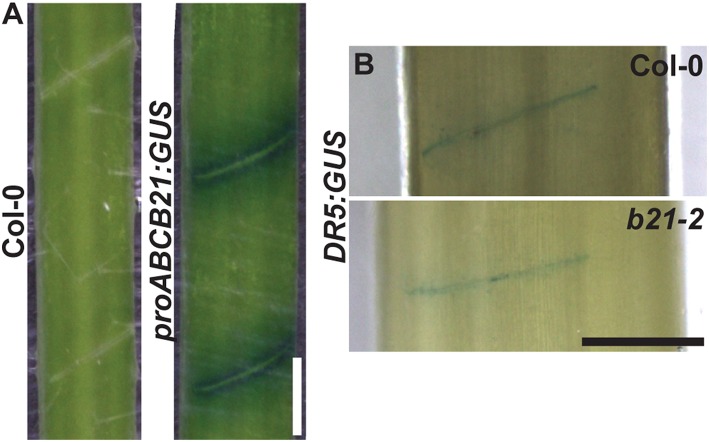
*proABCB21:GUS* and *DR5:GUS* expression after wounding. **(A)** *proABCB21:GUS* expression is induced after manual wounding. Mature inflorescence stems were cut with a razor blade at the bottom internode. Attached stems were left for 2 h prior to GUS staining. **(B)** Initial *DR5:GUS* expression is not different between Col-0 and *abcb21-2*. Plants were treated the same as in **(A)** except cleared with Visikol prior to imaging to enhance visualization. Scale bars: 0.5 mm.

As reported previously (Kamimoto et al., 2012), *proABCB21:GUS* expression in late stage mature tissues is restricted to the abscission zones of flowers, as well as rosette and cauline leaves (Figures 7A–E). Auxin regulation of leaf positioning (Peeters et al., 2009; de Carbonnel et al., 2010) and floral organ shedding/abscission (Tang et al., 2013) suggests a possible role for ABCB21 in regulation of localized auxin accumulations in these tissues. However, no differences in light-mediated leaf positioning were observed in *abcb21* mutants when responses under continuous 50 μmol m^−2^ s^−1^ red plus far red light, 50 μmol m^−2^ s^−1^ red light, or 50 μmol m^−2^ s^−1^ white light were examined (Figure 7F), and measurements of petal break-strength was not different between Col-0 and *abcb21-2* (Figure 7G). It is unclear whether *ABCB21* expression at these junction sites is responsive or causal. However, wounding increases *ABCB21* expression ~1.7X between 30 and 60 min before returning to pre-wound levels or below (Kilian et al., 2007). Rapid induction of *proABCB21:GUS* expression is observed in stem tissues after wounding (Figure 8A). No GUS staining was observed in Col-0 indicating staining was not due to non-specific enzymatic activity. However, similar discrete *DR5:GUS* signals are initially observed in both Col-0 and *abcb21-2* suggesting initial auxin accumulations are not affected (Figure 8B). A downstream role in wound-induced vascularization is possible, but does not appear to involve monolignol transport, as is observed with ABCG29 (Alejandro et al., 2012). No differences in seedling root growth on *p*-coumaryl alcohol were observed in *abcb21* under conditions where *abcg29* root growth is more inhibited than Col-0 (Supplementary Table 1), and no differences in lignin content or speciation were detected in seedling roots (Supplementary Table 2). A more localized impact on auxin-dependent vascularization is possible, but could not be reproducibly verified.

## Discussion

**Figure 9:**
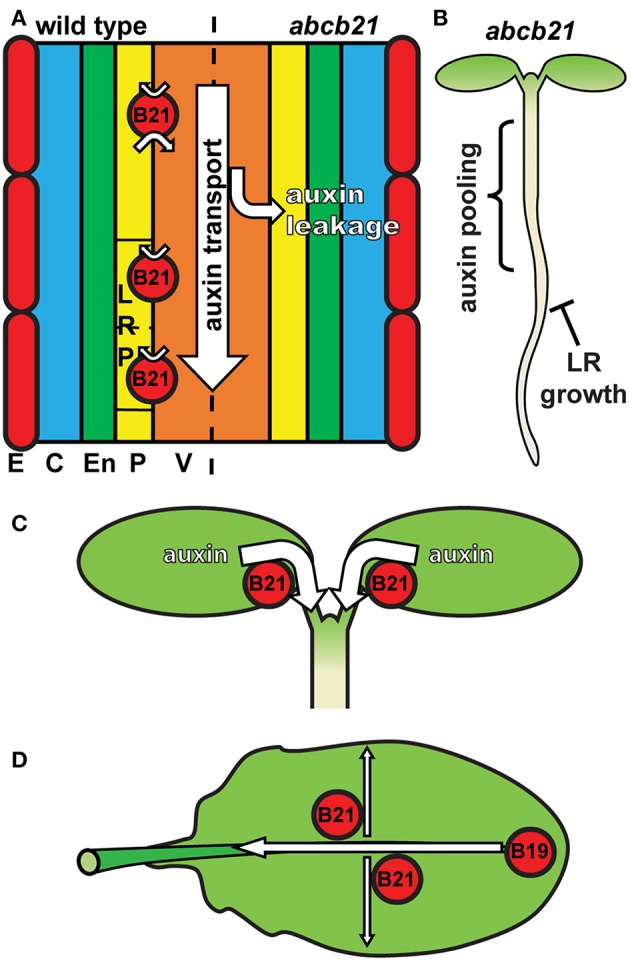
Model for ABCB21 function in seedlings and leaves. **(A)** ABCB21 functions primarily in restricting auxin to within the rootward auxin transport stream. Loss of ABCB21 leads to leakage of auxin from the central cylinder, resulting in reduced rootward auxin transport. E, epidermis; C, cortex; En, endodermis; P, pericycle; V, vasculature. **(B)** The reduction in auxin transport in the root causes pooling of auxin in the upper root and lower hypocotyl, reducing the supply needed for lateral root outgrowth. **(C)** ABCB21 mediates cotyledon-hypocotyl auxin transport to load the rootward auxin transport stream. **(D)** In leaves ABCB21 contributes to lateral auxin transport, while ABCB19 primarily mediates transport from the leaf tip to the petiole.

The results presented herein are consistent with a role for ABCB21 in regulating cellular auxin levels in a manner similar to ABCB4 in the root epidermis. In roots, *ABCB21* expression in the pericycle up to 7 d coincides with the rootward pulse of shoot derived auxin that triggers lateral root outgrowth (Bhalerao et al., 2002). *abcb21* mutants exhibit reduced rootward auxin transport and delays in lateral root emergence, which is consistent with the activity of ABCB19 (Wu et al., 2007). This suggests that in the root pericycle not associated with lateral roots ABCB21 primarily plays a supplementary role to ABCB19 in excluding auxin from the pericycle and maintaining it within the central cylinder (Figure 9A). Although ABCB21 exhibits conditional uptake/efflux activity, the endogenous auxin levels in the seedling root suggests ABCB21 is primarily acting in efflux in these tissues. The defects in lateral root emergence are likely due primarily to the defects in rootward auxin transport (Figure 9B). However, the loss of *ABCB21* expression in developing and emerging lateral roots suggests ABCB21 may also play a more localized role during lateral root development. Since ABCB21 exhibits auxin uptake activity at low intracellular concentrations, ABCB21 may help regulate initial auxin accumulations during the early stages of lateral root development. Expression of *ABCB21* during the first cell divisions during early lateral root development is consistent with this function. Additionally, treatment of lateral root primordia with cytokinins delays the establishment of auxin maxima and subsequent lateral root outgrowth (Bielach et al., 2012). These delays are also observed in *abcb21* suggesting cytokinins may negatively regulate *ABCB21* to allow for development of auxin maxima and progression of lateral root development after initiation. Loss of *ABCB21* expression during late stage development and emergence could be expected, as preventing reloading of auxin back into the rootward stream seems necessary for the establishment of auxin maxima at the primordia tip (Benková et al., 2003).

In cotyledons *ABCB21* expression coincides with the timing of shoot-derived auxin production (Bhalerao et al., 2002). *abcb21* mutants exhibit reduced cotyledon-hypocotyl auxin transport, increased cotyledon expansion, and decreased phototropic bending. These results reflect a role for ABCB21 in mobilizing auxin from the cotyledons to load the rootward auxin transport stream during the 5-10 d window when the cotyledons supply auxin to the root (Bhalerao et al., 2002) (Figure 9C). It was hypothesized that that loss of *ABCB21* would result in decreased transport from the leaf tip to the petiole. However, this resulted in reduced lateral auxin transport, not reduced transport from the tip to the petiole. While the precise cellular transport mechanisms remain to be determined, these results support a role for ABCB21 in contributing to auxin lateral distribution within the leaf, while ABCB19 primarily contributes to transport from the leaf tip to the petiole (Figure 9D). This is further supported by the observed alterations in leaf morphology and reduced epidermal cell size in *abcb1 abcb19 abcb21* triple mutants compared to *abcb1 abcb19* double mutants.

The role of ABCB21 in rosette leaf and floral organ abscission zones remains unclear. Treatment with auxin, salicylic acid, or methyl-jasmonate does not induce *ABCB21* expression (Kamimoto et al., 2012). However, H_2_O_2_ and UV-B, presumably by UV-induced reactive oxygen species (ROS), increases *ABCB21* expression ~1.9X and ~6X, respectively (Kilian et al., 2007; Gutiérrez et al., 2014). During wounding, increased ROS levels at the wound site within minutes (L'Haridon et al., 2011; Beneloujaephajri et al., 2013) correlates with the rapid induction of *ABCB21* expression. However, no difference in auxin accumulations or auxin related phenotypes are observed in these tissues, suggesting a role for ABCB21 in the transport of other substrates besides auxin.

ABCB4 and ABCB21 share high protein sequence similarity (83.8% identity/92.4% similarity) (Supplementary Figure 5). Previous analysis of protein sequence and structure identified an N-terminal coiled-coil domain that is present in ABCB4 and ABCB21, but not ABCB1 or ABCB19 (Yang and Murphy, 2009). The function of this domain remains unknown, but appears to be unique to ABCBs associated with substrate uptake in addition to efflux (Shitan et al., 2003; Santelia et al., 2005; Terasaka et al., 2005; Lee et al., 2008; Yang and Murphy, 2009; Kubeš et al., 2012). Although ABCB4 and ABCB21 function in a similar manner at the cellular level, compensatory activity that is observed with ABCB1/19 and ABCB6/20 (Noh et al., 2001; Zhang et al., 2018) is not present. This lack of functional redundancy is explained by the non-overlapping expression domains and points to involvement in distinct developmental processes. ABCB21 represents the final complement to the primary ABCB auxin transporter pairs. Therefore, ABCB1/19, ABCB6/20, and ABCB4/21 appear to represent the major ABCB auxin transporters in Arabidopsis.

## Data Availability

This manuscript contains previously unpublished data. The name of the repository and accession number are not available.

## Author Contributions

MJ, NC, and AM designed the research. MJ, NC, CP, and AM performed the experiments and analyzed the data. MJ and AM wrote the manuscript.

### Conflict of Interest Statement

The authors declare that the research was conducted in the absence of any commercial or financial relationships that could be construed as a potential conflict of interest.
