# Corrigendum: The Arabidopsis ATP-BINDING CASSETTE Transporter ABCB21 Regulates Auxin Levels in Cotyledons, the Root Pericycle, and Leaves

**DOI:** 10.3389/fpls.2020.00351

**Published:** 2020-04-09

**Authors:** Mark K. Jenness, Nicola Carraro, Candace A. Pritchard, Angus S. Murphy

**Affiliations:** ^1^Department of Plant Science and Landscape Architecture, University of Maryland, College Park, MD, United States; ^2^Department of Horticulture and Landscape Architecture, Purdue University, West Lafayette, IN, United States

**Keywords:** ABCB transporter, Arabidopsis thaliana, auxin, development, seedling

In the original article, there was a mistake in Figure 7F as published. The red light source used was subsequently found to emit a small amount of far-red. The experiments were repeated with a light setup that eliminated all far-red spectra. Under these conditions, abcb21 mutants were still not different from Col-0. A correction has been made to Figure 7, its legend and the Results section.

**Figure 7:**
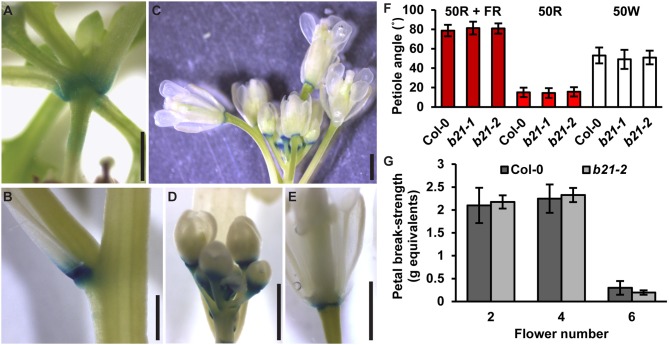
Expression of *ABCB21* in abscission zones. *proABCB21:GUS* is expressed in the abscission zones of **(A)** rosette leaves, **(B)** cauline leaves, and **(C)** floral organs. **(D,E)** *proABCB21:GUS* expression domain is expanded in **(D)** young flowers and **(E)** restricted to the abscission zone in mature flowers. Plants were GUS stained for 16 h. **(F)** True leaf petiole angles in *abcb21*. Plants were grown on soil under 80 μmol m^−2^ s^−1^ white light, 16 h photoperiod. When plants reached stage 1.01 they were transferred to continuous 50 μmol m^−2^ s^−1^ red plus far-red light (50R + FR; burgundy bars), 50 μmol m^−2^ s^−1^ red light (50R; red bars), or 50 m^−2^ s^−1^ white light (50W; white bars) and allowed to grow an additional 3 d. Angle was determined by measuring the angle formed between the hypocotyl and the two first true leaf petioles minus 90°. Data shown are means ± SD (*n* = 60). **(G)** Flower petal break-strength in *abcb21*. Flower 1 was designated as the first flower with visible flower petals. Methods are detailed the methods section and **Supplementary Figure 7**. Data shown are means ± SD (*n* = 15). Scale bars: 1 mm.

The Results section, subsection *ABCB21* Expression Is Rapidly Induced During Wounding:

“As reported previously (Kamimoto et al., 2012), *proABCB21:GUS* expression in late stage mature tissues is restricted to the abscission zones of flowers, as well as rosette and cauline leaves (Figures 7A–E). Auxin regulation of leaf positioning (Peeters et al., 2009; de Carbonnel et al., 2010) and floral organ shedding/abscission (Tang et al., 2013) suggests a possible role for ABCB21 in regulation of localized auxin accumulations in these tissues. However, no differences in light-mediated leaf positioning were observed in *abcb21* mutants when responses under continuous 50 μmol m^−2^ s^−1^ red plus far red light, 50 μmol m^−2^ s^−1^ red light, or 50 μmol m^−2^ s^−1^ white light were examined (Figure 7F), and measurements of petal break-strength was not different between Col-0 and *abcb21-2* (Figure 7G). It is unclear whether *ABCB21* expression at these junction sites is responsive or causal. However, wounding increases *ABCB21* expression ~1.7X between 30 and 60 min before returning to pre-wound levels or below (Kilian et al., 2007). Rapid induction of *proABCB21:GUS* expression is observed in stem tissues after wounding (**Figure 8A**). No GUS staining was observed in Col-0 indicating staining was not due to non-specific enzymatic activity. However, similar discrete *DR5:GUS* signals are initially observed in both Col-0 and *abcb21-2* suggesting initial auxin accumulations are not affected (**Figure 8B**). A downstream role in wound-induced vascularization is possible, but does not appear to involve monolignol transport, as is observed with ABCG29 (Alejandro et al., 2012). No differences in seedling root growth on *p*-coumaryl alcohol were observed in *abcb21* under conditions where *abcg29* root growth is more inhibited than Col-0 (**Supplementary Table 1**), and no differences in lignin content or speciation were detected in seedling roots **(Supplementary Table 2)**. A more localized impact on auxin-dependent vascularization is possible, but could not be reproducibly verified.”

The authors apologize for this error and state that this does not change the scientific conclusions of the article in any way. The original article has been updated.
