# Supplementary material for: The Arabidopsis ATP-BINDING CASSETTE Transporter ABCB21 Regulates Auxin Levels in Cotyledons, the Root Pericycle, and Leaves
Source: Front Plant Sci. 2019 Jun 19;10:806. doi: 10.3389/fpls.2019.00806 (PMC6593225; doi:10.3389/fpls.2019.00806)
Supplement: Supplementary file 2 [file Data_Sheet_1.docx]

Supplementary Material

## Supplementary Figure 1. Expression in *abcb* mutants.

## Supplementary Figure 2. NPA inhibition of auxin transport in leaves.

## Supplementary Figure 3. Rosettes of single and double *abcb4* and *abcb21* mutants.

## Supplementary Figure 4. Inflorescence phenotypes of *abcb4* and *abcb21*.

## Supplementary Figure 5. ABCB4/21 protein alignment.

## Supplementary Figure 6. Description of measurement for phototropism assays.

## Supplementary Figure 7. Petal break-strength setup.

**Supplementary Table 1.** Primary root lengths (cm) of 14 d seedlings grown on medium supplemented with *p*-coumaryl alcohol.

**Supplementary Table 2.** Contents of lignin thioacidolysis products in *abcb21.*

## Supplementary Table 3. Lines used in this study.

## Supplementary Table 4. Primers used in this study.

## Supplementary Movie 1. Phototropic bending of Col-0 and *abcb21-2*.


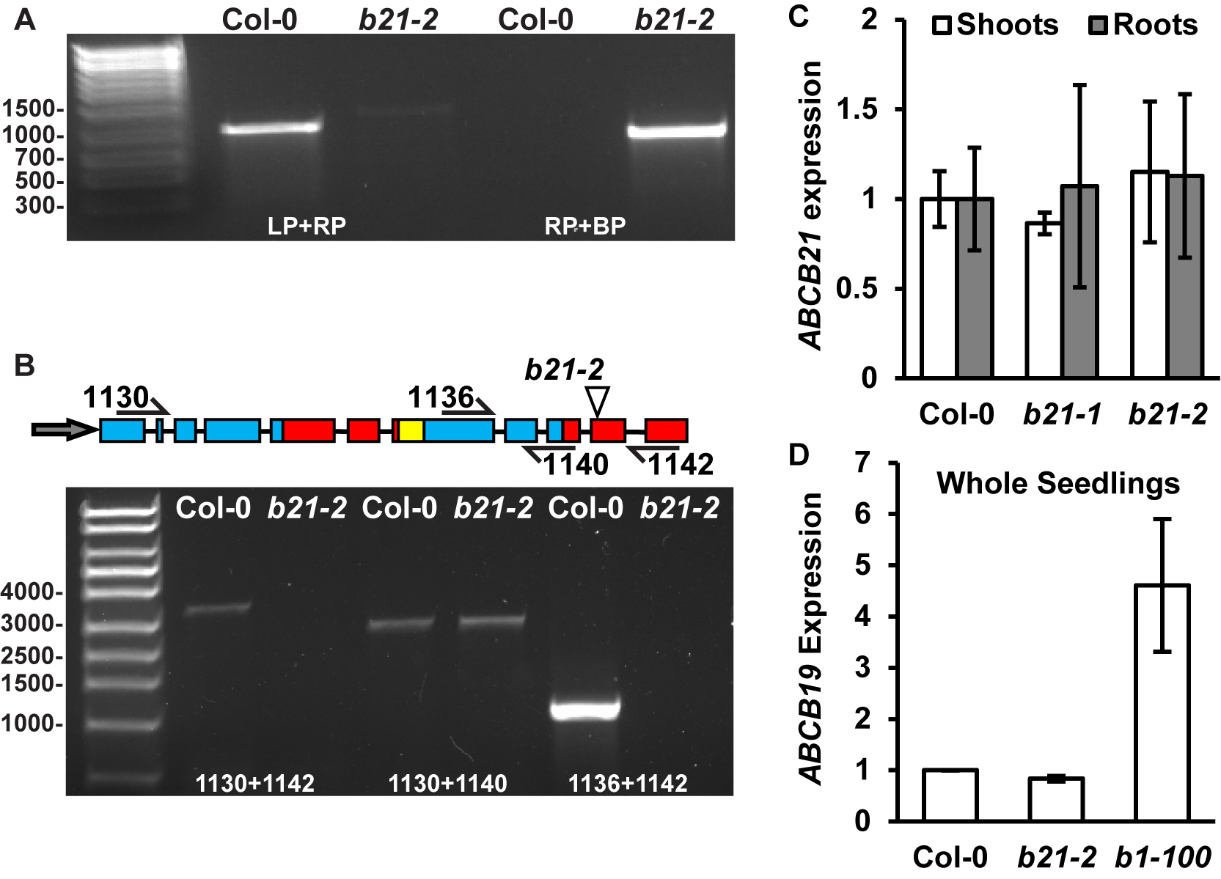


**Supplementary Figure S1. Expression in *abcb* mutants. (A)** PCR analysis showing a 1131 bp wild-type band using LP and RP primers, and an *abcb21-2* T-DNA insert specific band using BP and RP primers. **(B)** PCR analysis of transcript length in *abcb21-2*. Approximate primer positions are indicated in the gene model. Similar results were obtained from two biological and two technical replicates. **(C)** Quantitative real-time PCR showing *ABCB21* expression in *abcb21-1* and *abcb21-2*. Data shown are means ± SD (n = 3 biological replicates, 2 technical replicates). **(B)** *ABCB19* expression in Col-0, *abcb21-2,* and *abcb1-100*. Data shown are means ± SD (n = 3 biological replicates, 2 technical replicates).

##
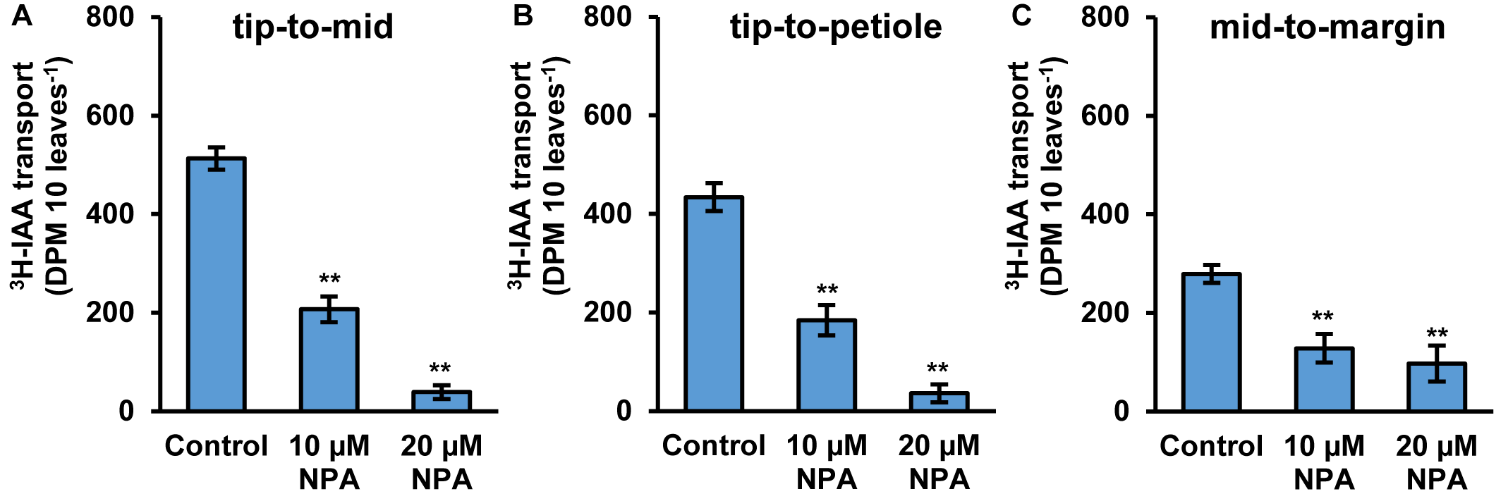


## Supplementary Figure 2. NPA inhibition of auxin transport in leaves. Transport of [^3^H]IAA from the (A) leaf tip to the leaf midpoint or (B) leaf tip to the leaf petiole, or (C) from the leaf midvein to the margin following treatment with 10 and 20 µM NPA. Intact leaves of 35 d were emerged in NPA prior to [^3^H]IAA application with agarose beads. Excess NPA solution was allowed to drip off and remaining droplets removed by lightly dabbing with a cotton swab. Data shown are means ± SD (n = 3 pools of 10). Asterisks indicate statistical difference from Col-0 by Student’s t test for * *P* < 0.05 and ** *P* < 0.01.


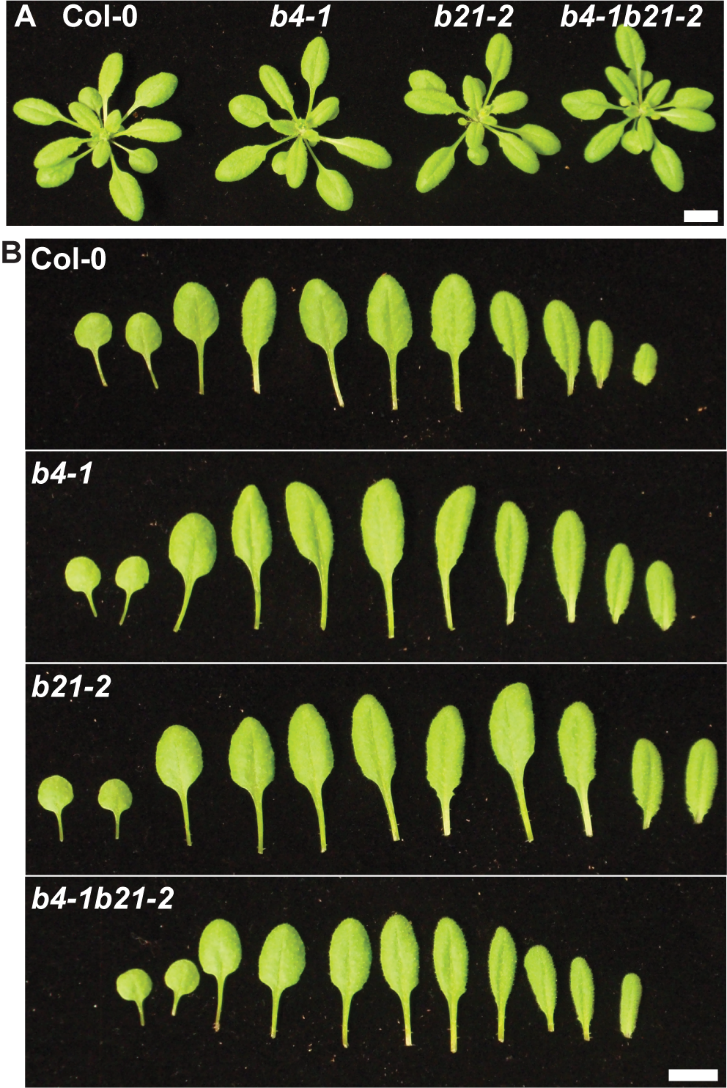


**Supplementary Figure S3. Rosettes of single and double *abcb4* and *abcb21* mutants.** **(A)** Representative images of 21 d rosettes. **(B)** Rosette leaves from (A).Representative images of rosette leaves from 21 d *abcb4-1*, *abcb21-2*, and *abcb4-1 abcb21-2* mutants. Scale bars: 1 cm.


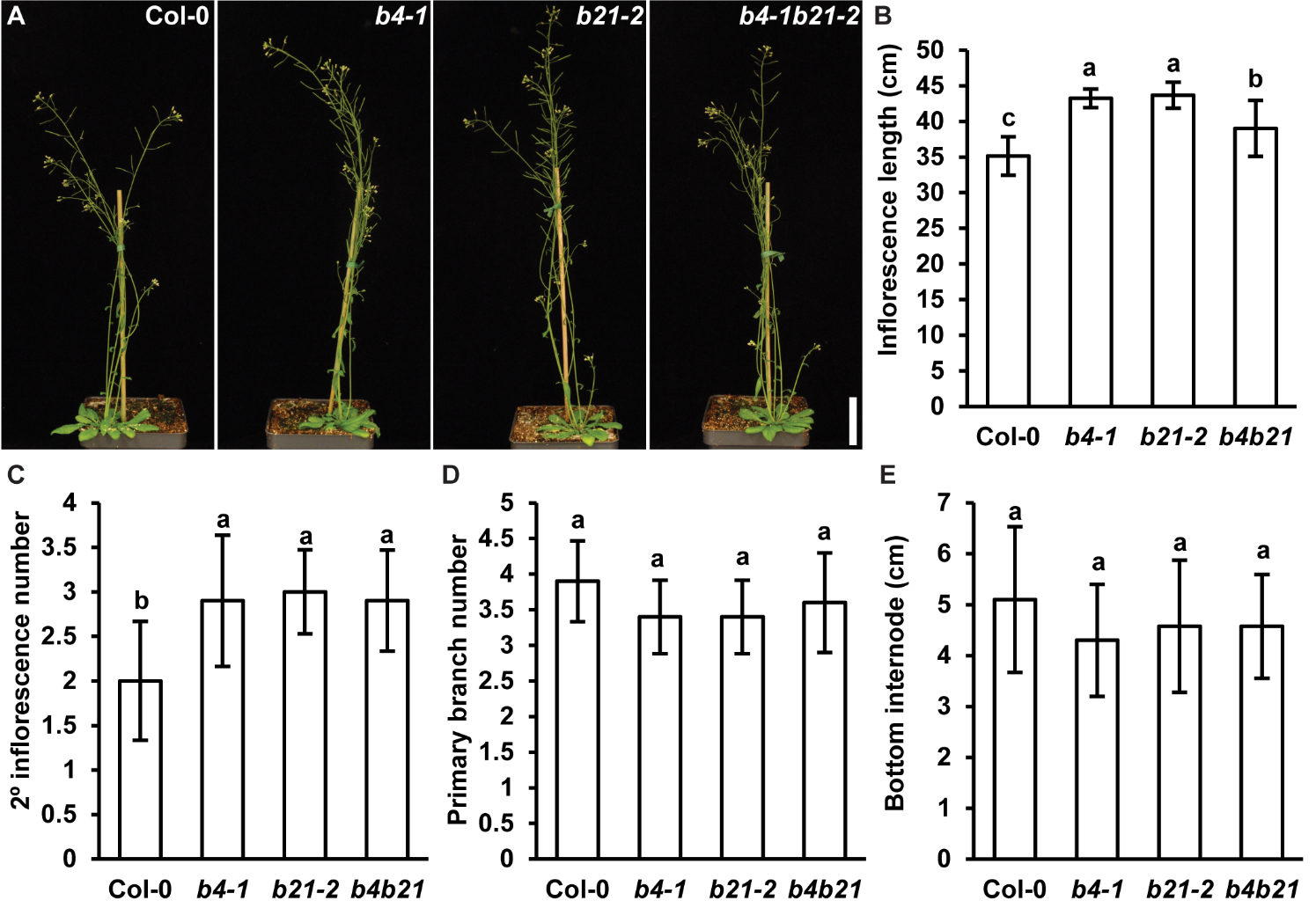


**Supplementary Figure S4. Inflorescence phenotypes of *abcb4* and *abcb21*.** **(A)** Representative images of 35 d inflorescences. Measurement of **(B)** primary inflorescence length, **(C)** secondary (2°) inflorescence number, **(D)** primary branch number, and **(E)** internode length in 35 d plants. Data shown are means ± SD (n = 10). Lowercase letters indicate statistical difference by ANOVA *P* < 0.001, Tukey’s post-hoc *P* < 0.05. Scale bar: 5 cm.

**
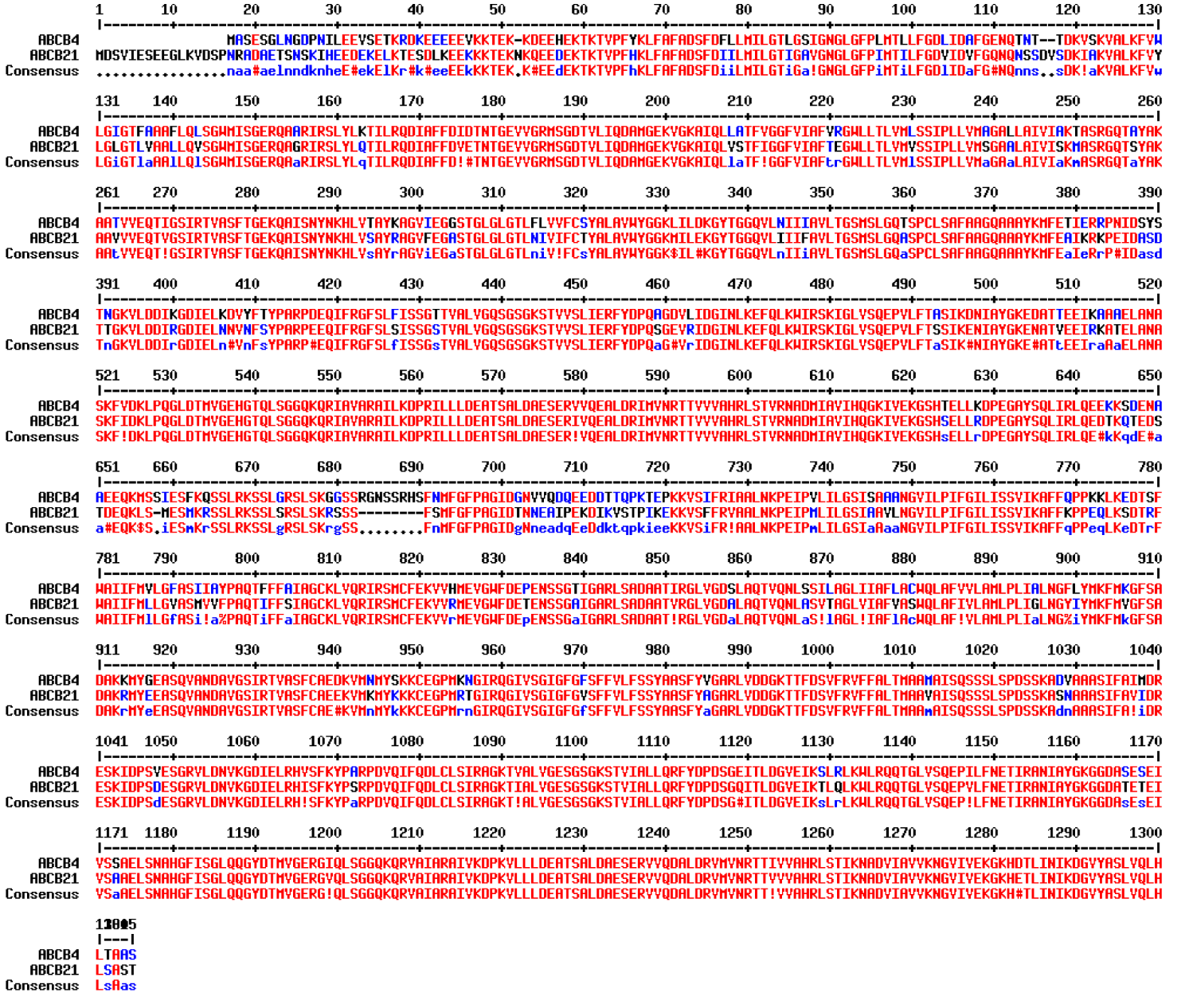
**

**Supplementary Figure S5. ABCB4/21 protein alignment.** ABCB4 and ABCB21 share 83.8% identity and 92.4% similarity. Noteworthy differences are only found with the N-terminal and linker regions. Sequence alignment was generated with MultAlin.


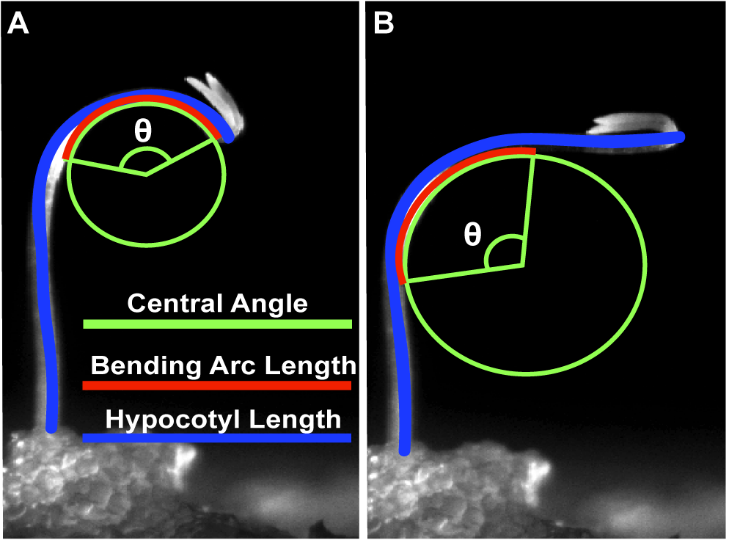


**Supplementary Figure S6. Description of measurement for phototropism assays. (A)** Assays conducted in open-air demonstrate that seedlings bend along a circular arc, thus the central angle can be extrapolated from the arc length and area of the circle that fits into the arc. Measurement of this central angle allows for isolation of the phototropic bending response **(A)** from hypocotyl elongation that occurs after the initial bending response **(B)**. This is especially useful for mutants that show hypocotyl elongation differences, since there is no anchoring of the angle measurement to any assumed fixed hypocotyl point not part of the visible bending arc area. Assuming all seedlings are selected for a uniform height, arc length can be used to describe the scope of the elongation zone along the hypocotyl. The extent to which this elongation zone migrates down the length of the hypocotyl can additionally be quantitated as the ratio of bending zone to overall hypocotyl length (arc/length ratio).


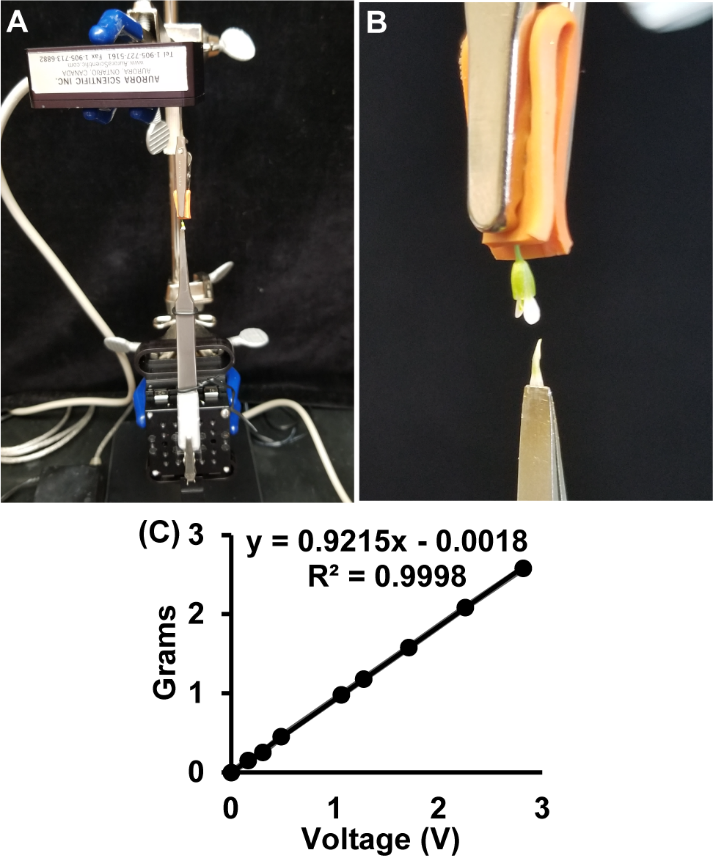


**Supplementary Figure S7. Petal break-strength setup.** **(A)** The petal break-strength setup consisted of a force sensor, a flower holder, flat ended forceps, and a translation stage. **(B)** Vertical displacement of the stage resulted in reproducible detachment of the petal at the receptacle. **(C)** Measurements were converted to gram equivalents according to a linear standard curve (voltage as a function of weight) corrected for the weight of the clip.

**Supplementary Table S1. Primary root lengths (cm) of 14 d seedlings grown on medium supplemented with *p*-coumaryl alcohol.**

| **Root length (cm) of 14 d seedlings grown on medium containing *p*-coumaryl alcohol** | | | |
| --- | --- | --- | --- |
|  | **Control** | **1.5 mM** | **2 mM** |
| Col-0 | 5.9 ± 0.41 | 3.9 ± 0.23 | 2.3 ± 0.45 |
| *abcg29-1* | 5.7 ± 0.85 | 2.8 ± 0.33 | 1.8 ± 0.38 |
| *abcb4-1* | 5.1 ± 0.93 | 3.3 ± 0.52 | 1.9 ± 0.24 |
| *abcb21-1* | 6.0 ± 0.74 | 4.1 ± 0.29 | 2.1 ± 0.30 |

Seedlings were grown on ¼ MS, 0.5% sucrose, 0.8% agar (pH 5.5) medium supplemented with 1.5 or 2 mM *p*-coumaryl alcohol. Seedlings were grown under 140 µmol m^-2^ s^-1^ with a 15 h photoperiod. Data shown are means ± SD (n = 10).

**Supplementary Table S2. Contents of lignin thioacidolysis products in *abcb21.***

| **Lignin content (nmol g^-1^ FW)** | | | |
| --- | --- | --- | --- |
|  | ***p*-hydroxyphenyl** | **guaiacyl** | **syringyl** |
| Col-0 | 10.3 ± 0.9 | 10.3 ± 0.9 | 10.3 ± 0.9 |
| *abcb21-1* | 11.7 ± 0.7 | 11.7 ± 0.7 | 11.7 ± 0.7 |

Data represent means ± SE for 3 replicates of 30 seedling roots. Lignin contents were determined by GC-MS as described (Alejandro et. al., 2012).

**Supplementary Table S3. Lines used in this study.**

| **Gene Locus** | **Protein** | **Mutant** | **T-DNA Insertion** | **Reference** |
| --- | --- | --- | --- | --- |
| AT2G36910 | ABCB1 | *abcb1-100* | Salk_083649 | Lin and Wang, 2005 |
| AT2G47000 | ABCB4 | *abcb4-1* | Salk_063720 | Terasaka et al., 2005 |
| AT3G28860 | ABCB19 | *abcb19-101* | Salk_033455 | Lin and Wang, 2005 |
| AT3G62150 | ABCB21 | *abcb21-1* | WiscDsLox1C2 | Kamimoto et al., 2012 |
|  |  | *abcb21-2* | Gabi_954H06 | This study |
| AT3G16340 | ABCG29 | *abcg29-1* | Salk_081047 | Alejandro et al., 2012 |

**Supplementary Table S4. Primers used in this study.**

| **Quantative real-time PCR** | | |
| --- | --- | --- |
| **Gene** | **5’-Sequence-3’** | **Sense** |
| *ABCB1* | TCTCTTGCTTTGGTACGGTG  CTCGGTGCTGATTGTCCC | For.  Rev. |
| *ABCB19* | AGGATTGACCCGGATGATGCTGAT  TCGGGTCTTGAAGGGTAAGCGAAA | For.  Rev. |
| *ABCB21*  (Kamimoto et al., 2012) | TCGCTCATACGTCTACAAGAAGATACTAAACAG  CGAAAGAGACTTTCTTTTCTTTGATCGG | For.  Rev. |
| *PP2A* | TCGTGGTGCAGGCTACACTTTC  TCAGAGAGAGTCCATTGGTGTGG | For.  Rev. |
| *ACT2* | ACACTGTGCCAATCTACGAGGGTT  ACAATTTCCCGCTCTGCTGTTGTG | For.  Rev. |
| **Promoter:GUS Constructs** | | |
| **Construct** | **5’-Sequence-3’** | **Sense** |
| *ABCB21* | CACCAATTGTAAAGAAAAAGTTATGAGTC  TGTTCTTTGATCCTATCAAGA | For.  Rev. |
| **Expression in *S.pombe*** | | |
| *ABCB21* | ggggacaagtttgtacaaaaaagcaggcttcatggatagtgtaatagaatcagag  ggggaccactttgtacaagaaagctgggtcctattatgtagaagcactcagatgaagttg | For.  Rev. |
| **Genotyping** | | |
| *abcb1-100* | GAAGACTGCGACAAGGACAAG  GCAAGAGCGATGTTGAAGAAC | For.  Rev. |
| *abcb4-1* | TGTGGATAAGCTACCACAGGG  AAATGCAATGATCAAACCAGC | For.  Rev. |
| *abcb19-101* | GCAATTGCAATTCTCTGCTTC  CTCAGGCAATTGCTCAAGTTC | For.  Rev. |
| *abcb21-1* | AATCGACAGTGATTGCGTTG  TTAACCATAACCCGGTCCAA | For.  Rev. |
| *abcb21-2* (LP)  (RP) | TTCTCCACGATGACTCCATTC  TCATTGTCTCCTGATTCCAGC | For.  Rev. |
| p745 | AACGTCCGCAATGTGTTATTAAGTTGTC | T-DNA |
| o8409 (BP) | ATATTGACCATCATACTCATTGC | T-DNA |
| ***abcb21-2* transcript analysis** | | |
| 1130 | AAGATCGCTAAGGTGGCTCTG | For. |
| 1136 | TATAGCCGCGGTCTTAAACGGAG | For. |
| 1140 | CCAGACTCATCACTTGGATC | Rev. |
| 1142 | TGGCTTCATCAAGTAGCAACAC | Rev. |

**Supplementary Video 1. Phototropic bending of Col-0 and *abcb21-2*.** *abcb21* exhibits defects in phototropism in light-treated seedlings. Seedlings 1-4 are *abcb21-2* and seedlings 5-8 are Col-0. Video timeframe is the 3 h period represented in Fig. 4H.
